# A systematic study of motif pairs that may facilitate enhancer–promoter interactions

**DOI:** 10.1515/jib-2021-0038

**Published:** 2022-02-07

**Authors:** Saidi Wang, Haiyan Hu, Xiaoman Li

**Affiliations:** Department of Computer Science, University of Central Florida, Orlando, FL, 32816, USA; Burnett school of Biomedical Science, College of Medicine, University of Central Florida, Orlando, FL, 32816, USA

**Keywords:** enhancer–promoter interactions, enhancers, motif pairs, promoters, transcription factors

## Abstract

Pairs of interacting transcription factors (TFs) have previously been shown to bind to enhancers and promoters and contribute to their physical interactions. However, to date, we have limited knowledge about such TF pairs. To fill this void, we systematically studied the co-occurrence of TF-binding motifs in interacting enhancer–promoter (EP) pairs in seven human cell lines. We discovered 423 motif pairs that significantly co-occur in enhancers and promoters of interacting EP pairs. We demonstrated that these motif pairs are biologically meaningful and significantly enriched with motif pairs of known interacting TF pairs. We also showed that the identified motif pairs facilitated the discovery of the interacting EP pairs. The developed pipeline, EPmotifPair, together with the predicted motifs and motif pairs, is available at https://doi.org/10.6084/m9.figshare.14192000. Our study provides a comprehensive list of motif pairs that may contribute to EP physical interactions, which facilitate generating meaningful hypotheses for experimental validation.

## 1 Introduction

Identifying enhancer–promoter (EP) interactions is important for the understanding of gene transcriptional regulation [1]. Enhancers are short genomic regions that can strengthen their target genes’ transcriptional levels independent of their distance and orientation to the target genes [2]. They are in general several hundred base pairs (bps) long, can be hundreds to thousands of bps away from their target genes, and can be in the upstream or downstream of the target genes or introns. By interacting with promoters of their target genes, enhancers increase target genes’ transcription and modulate their condition-specific expression [2–4].

Many studies have attempted to identify EP interactions. Experimental approaches based on chromatin conformation capture techniques and their extensions have identified many EP interactions across several cell lines, cell types and tissues [1, 5], [6], [7], [8], [9], [10], [11]. These experimental approaches nurtured our rudimentary understanding of EP interactions. However, they are either time-consuming or still costly because of the large number of EP interactions under an experimental condition and the required high-sequencing depth to comprehensively identify them on the genome scale [1, 12]. Computational methods for EP interaction predictions are thus indispensable. These methods usually consider the distance, conservation, correlated activity between enhancers and promoters, etc., to identify EP interactions [13–22]. Although having shown success, they have a suboptimal performance on discovering EP interactions, especially condition-specific EP interactions [17, 23], [24], [25], [26]. It is thus necessary to further investigate the characteristics of EP interactions, which may significantly improve the accuracy of the existing methods.

Several studies pointed out a new venue to explore the characteristics of EP interactions, which suggested that the interaction of transcription factors (TFs) that bind an enhancer and TFs that bind a promoter of an EP pair may contribute to the interaction of this EP pair [2, 12, 19, 22, 27], [28], [29], [30]. For instance, it is well known that the TF and structural protein CTCF binds to a fraction of enhancers and promoters, which facilitates the physical interaction of enhancers and promoters in these EP pairs [31]. Another example, the ubiquitous TF YY1, binds to enhancers and promoters and contributes to EP interactions [32]. It is thus promising to systematically study the potential interactions of TFs that bind to enhancers and promoters and understand how such interactions may lead to the interaction of EP pairs. A computational study integrated chromatin immunoprecipitation followed by massive parallel sequencing (ChIP-seq) data and Hi-C data in two cell lines and predicted 565 interactions of DNA-binding proteins, including TFs [12]. This study was encouraging while limited to a small number of TFs in only two cell lines. To date, we still lack a clear view of the interaction of which TF pairs may render the specificity of the interaction of the enhancer and the promoter in an EP pair.

We systematically investigated the co-occurrence of potential TF binding motifs in enhancers and their corresponding interacting promoters (Material and Methods). A motif is a TF binding pattern, which is often represented by a position weight matrix [33, 34]. We identified 114 non-redundant motifs in interacting EP pairs that represented the binding patterns of potential TFs. We also identified 423 motif pairs that significantly co-occurred in interacting EP pairs. Interestingly, on average, more than 62% of these motif pairs in a cell line were shared across cell lines and were able to help to distinguish true interacting EP pairs from false ones. Our study provides a comprehensive list of motif pairs that may contribute to EP physical interactions and facilitate their predictions, which also creates meaningful hypotheses for experimental validation of EP interactions.

## 2 Methods

### 2.1 Positive and negative EP pairs

We downloaded the Hi-C contact matrices in the following seven cell lines: GM12878, HMEC, HUVEC, IMR90, K562, KBM7 and NHEK, which were normalized with the Knight and Ruiz normalization vectors by Rao et al. [1]. We claimed that two genomic regions interacted in a cell line (except GM12878) if the corresponding entry in the normalized contact matrix of this cell line was larger than 30. The interacting regions defined by this cutoff would include almost all pairs of interacting regions defined in IMR90 and K562 by independent studies [6, 7]. Because the Hi-C sequencing depth in GM12878 was one magnitude larger than that in all other cell lines (Supplementary S1), to control false positives, we used a larger cutoff of 150 in GM12878. This larger cutoff resulted in a similar number of selected pairs of interacting regions in GM212878 [1]. In this way, we had positive pairs of interacting regions. Note that we could use the looplists defined by Rao et al. as positive pairs of interacting regions [1]. However, the number of looplists was small, which resulted in an even smaller number of positive EP pairs that could not be used to discover interacting TF pairs below.

**Figure 1:**
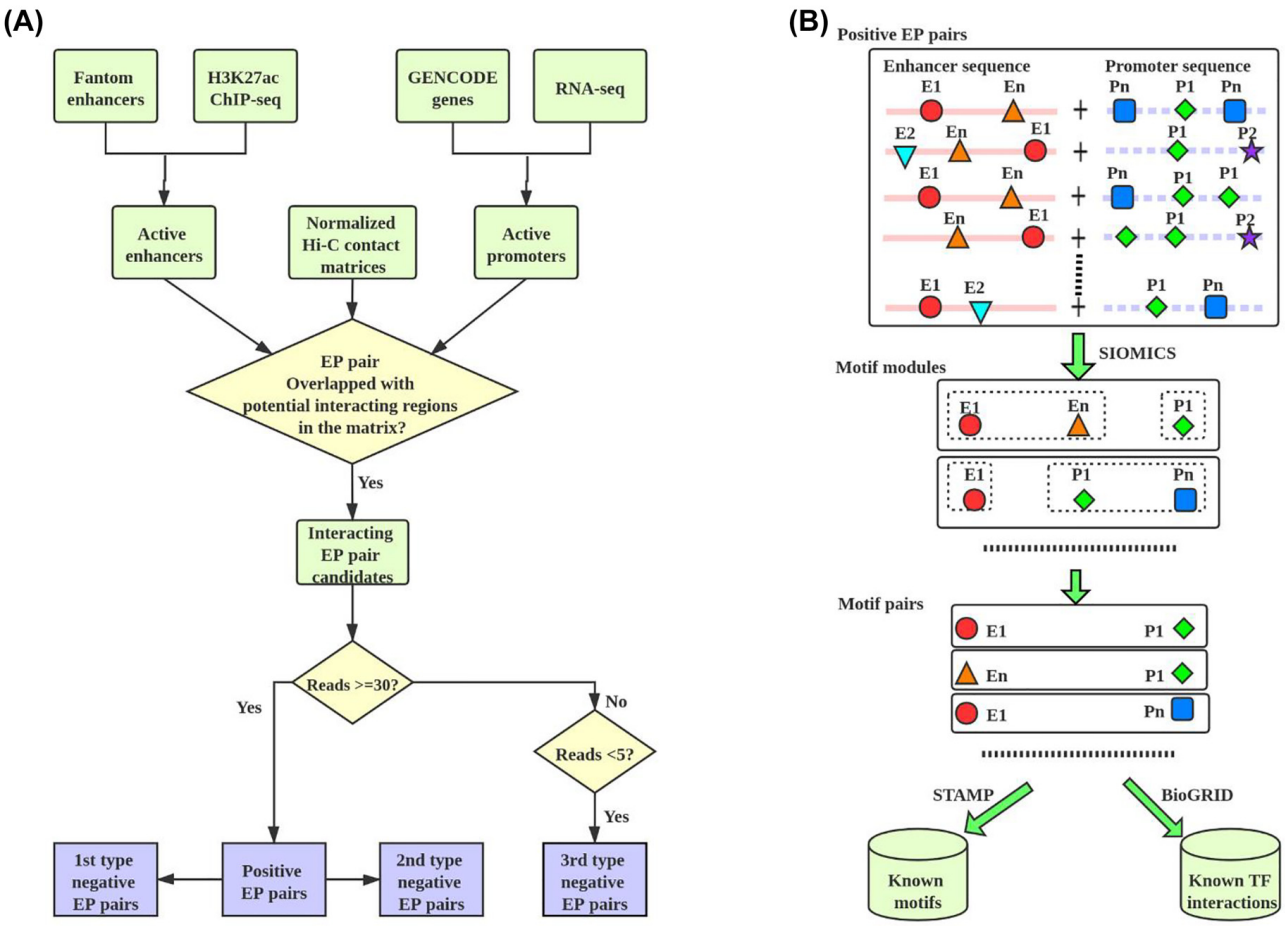
(A) The procedure to obtain positive and negative EP pairs. (B) The pipeline to study motif pairs in positive EP pairs.

To obtain positive EP pairs in a cell line, we overlapped the above positive pairs of genomic regions with the corresponding “active” enhancers and “active” promoters (Figure 1A). An active enhancer was one of the 32,284 enhancers defined by FANTOM [35] that overlapped with the H3K27ac ChIP-seq peaks [36] in the corresponding cell line. To our knowledge, FANTOM enhancers were the largest collection of mammalian enhancers with direct experimental evidence. With the transcription start sites (TSSs) defined in GENCODE, we defined 57,820 promoters, each of which was the genomic region from the upstream 1000 bps to the downstream of 100 bps the TSS of a GENCODE gene. An active promoter was then defined with these GENCODE promoters and the ENCODE RNA-seq data as previously [22, 24]. In this way, every positive EP pair had its enhancer overlapping with one genomic region and its promoter overlapping with the other genomic region of a positive pair of genomic regions, and the distance between the active enhancer and the active promoter was within 2.5 kilobase pairs to 2 megabase pairs (Supplementary S1). The majority of the positive EP pairs were likely to be true positives, despite false positives and negatives.

To assess how well the predicted motif pairs facilitate the identification of true interacting EP pairs, we generated three types of negative EP pairs (Figure 1A and Supplementary S1). The first type was the permuted version of the positive ones. The enhancer and the promoter sequence of a negative EP pair were a random permutation of the enhancer and the promoter sequence in the corresponding positive EP pair, respectively. The second type of negative EP pairs was generated by replacing the enhancers with randomly chosen genomic regions in positive EP pairs. These random genomic regions had a similar length distribution and a similar distance distribution to promoters as the enhancers in positive EP pairs. The third type was defined from the normalized Hi-C contact matrices with the cutoff 5, similar to the positive EP pairs (Figure 1A). In brief, if a pair of genomic regions had fewer than five supported normalized Hi-C reads, we called this pair of regions a negative pair of genomic regions. We then overlapped the negative pairs of genomic regions with the active FANTOM enhancers and active GENCODE promoters to obtain negative EP pairs. The first two types of negative EP pairs were used to assess whether the predicted motif pairs could distinguish non-EP pairs from positive EP pairs, while the third type was used to determine whether they could separate the interacting pairs from non-interacting pairs.

### 2.2 Known non-redundant motifs

We collected known TF binding motifs from JASPAR and CIS-BP databases [33, 37]. We compared every pair of motifs from these two sources with the tool STAMP [38]. As previously [39, 40], if two motifs had an STAMP similarity E-value smaller than 1E-05, we claimed they were similar. We obtained 649 non-redundant known motifs from the two sources by keeping only one motif in each group of similar motifs.

### 2.3 EP motif pair discovery pipeline, EPmotifPair

We developed a pipeline EPmotifPair to identify motif pairs that may facilitate EP interactions. Starting from the repeat masked sequences in the enhancer and promoter regions of interacting EP pairs under a given experimental condition, this pipeline outputs statistically significant motif pairs that may contribute to the physical interaction of enhancers and their promoters. Every output motif pair is composed one motif in the enhancers and the other motif in the promoters.

In brief, first, EPmotifPair applies the SIOMICS tool [39, 41] to the repeat-free concatenated sequences to identify motif modules under an experimental condition. A concatenated sequence consists of the enhancer sequence and the promoter sequence of an interacting EP pair. A motif module is a group of motifs whose binding sites significantly co-occur in input sequences. Biologically, a motif module mimics the group of motifs for one TF and its cofactor TFs, where this TF and its cofactor TFs bind to sequences to regulate a common group of genes. We applies SIOMICS here because it considers multiple co-occurring sequence patterns to identify motif modules and motifs, which significantly reduces false positive predictions compared with the strategy to predict individual TF motifs separately [34]. Moreover, it can *de novo* predict motifs, which thus does not depend on the limited number of known motifs available.

Next, this pipeline processes the identified motif modules to obtain motif pairs. With the predicted motif modules, this pipeline filters motif pairs that co-occur in only enhancers or only promoters. That is, it keeps all motif pairs in each motif module that have one motif in promoters and the other motif in enhancers of interacting EP pairs. The occurrence of a motif in enhancers and promoters is defined by the SIOMICS predicted motif instances. The kept motif pairs that are significantly overrepresented (Poisson clumping heuristic [42], corrected p-value < 0.01) in the input sequences are the final motif pairs, the TFs of which may be likely to interact and contribute to the interaction of the EP pairs (Figure 1B).

Finally, EPmotifPair compares the predicted motif pairs with known TF interactions. It compares the predicted motifs in motif pairs with the above non-redundant known motifs. A predicted motif was similar to a known motif if the STAMP E-value was smaller than 1E-5. The TF(s) corresponding to this known motif was considered to be the TF(s) bound to this predicted motif. With the corresponding TFs of the predicted motifs, this pipeline compares the predicted motif pairs with known TF pairs in the BioGrid database (see details in Section 2.5).

### 2.4 Homogeneous motif pairs

In addition to the above heterogeneous motif pairs, we consider homogeneous motif pairs. A homogenous motif pair is composed a pair of the same motif that significantly co-occurs in both enhancers and promoters of positive EP pairs. We apply two approaches to measure the significance of such a co-occurrence of the same motif to identify homogeneous motif pairs. In one way, assume there are *N* positive EP pairs, and *n* has such a motif in both enhancers and promoters (based on FIMO scan). Assume the average promoter and enhancer length is *l*_1_ and *l*_2_ in this cell line, respectively. Also, assume this motif occurs *x* times in these *N* EP pairs. We calculate the p-value as *pbinom(n, N, p)*, where $p=\frac{x}{N*\left(l_{1}+l_{2}\right)}$ , $pbinom\left(n,N,p\right)=\sum_{i=n}^{N}\frac{N!}{i!\left(N-i\right)!}p^{i}{\left(1-p\right)}^{N-i}$ . If this p-value is smaller than 0.01/*K*, where *K* is the number of the predicted motifs in this cell line, we claim that this motif forms a homogeneous motif pair. In the other way, assume this motif occurs in *x* of the *N* enhancers and *y* of the *N* promoters based on the FIMO scan. We calculate the p-value with the same formula but different $p=\frac{x*y}{N*N}$ . If this p-value is smaller than 0.01/*K*, we claim this motif is significant.

### 2.5 Enrichment analysis of the predicted EP motif pairs

We compared the predicted EP motif pairs with known motif pairs of interacting TFs. We collected directly and indirectly interacting TF pairs from BioGRID [43]. The direct TF interactions meant that two TFs physically interacted with each other. The indirect ones referred to pairs of TFs without direct interaction but directly interacting with a common third protein. There were 6820 direct and 120,277 indirect pairs of known TF interactions in BioGRID, which involved 1520 and 1207 TFs, respectively. We then assessed the statistical significance of the enrichment of the motif pairs of known interacting TFs in the predicted motif pairs in every cell line by hypergeometric testing. In brief, assume there were N TFs and M pairs of TFs in BioGrid, among which there were *m* pairs that involved *n* TFs in the predicted EP motif pairs in a cell line. We calculated the p-value of enrichment of motif pairs of known interacting TFs as $phyper\left(m,\frac{n\left(n-1\right)}{2},M,\frac{N\left(N-1\right)}{2}\right)$ , where $phyper\left(x_{1},y_{1},x_{2},y_{2}\right)=\sum_{k=x_{1}}^{\min\left(y_{1},x_{2}\right)}\frac{y_{1}!\left(y_{2}-y_{1}\right)!x_{2}!\left(y_{2}-x_{2}\right)!}{y_{2}!k!\left(x_{2}-k\right)!\left(y_{1}-k\right)!\left(y_{2}-x_{2}-y_{1}+k\right)!}$ for any non-negative integers *x*_1_, *y*_1_, *x*_2_ and *y*_2_. We also compared the predicted EP motif pairs with those predicted in a previous study, which predicted 298 pairs of TF interactions involved 61 TFs in GM12878 and 46 pairs of TF interactions involved 22 TFs in K562 [12].

### 2.6 Enhancer and promoter enriched motifs

We studied whether a predicted motif preferred to occur in enhancers or promoters. We assessed the statistical significance of a preference in two ways by the binomial testing, similar to what we did in analyzing the homogenous motif pairs. That is, we calculated the significance by considering the number of sequences only or both the number and the length of sequences.

### 2.7 Machine learning methods to distinguish positive from negative EP pairs

We studied how well the predicted motif pairs distinguish positive from negative EP pairs. We described each EP pair with a 4*n* + *1* vector, where four entries were for each of the *n* motif pairs, and one entry was for the positive or negative status. The four entries for a motif pair were the occurrence number of its motifs (based on FIMO) in the enhancer and promoter, respectively.

We applied the following four methods (https://scikit-learn.org/stable/), random forests, least absolute shrinkage and selection operator (lasso), decision tree, and support vector machines [44–47], to distinguish positive from negative EP pairs. We did 10-fold cross-validation to measure the performance of different methods. The four methods had similar F1 scores in separating positives from negatives. Because lasso selected a subset of the predicted motif pairs while achieved similar performance, we presented our study with lasso in this study.

## 3 Results

### 3.1 The predicted motif pairs were likely to be biologically meaningful

We applied the EPmotifPair pipeline to the interacting EP pairs in seven cell lines and identified 434 motif pairs (Table 1). These motif pairs were from the predicted motif modules, each of which contained two to five motifs. As mentioned above, a motif module is a statically significant group of co-occurring motifs, representing the motif combination of a TF and its cofactors [48]. The developed EPmotifPair pipeline, together with the predicted motifs, motif pairs, motif modules, and other information, is available at https://doi.org/10.6084/m9.figshare.14192000.

**Table 1:** The predicted motif pairs in seven cell lines.

Cell line (billion)	#Enhancers	#Promoters	#EP pairs	#Predicted motifs	#Predicted motif pairs
GM12878 (15.1)	2731	2171	3688	51 (76.47%)	233 (66.52%, 0.86%, 1.23E-14)
HMEC (1.1)	1761	1713	2157	33 (87.88%)	88 (59.09%, 2.27%, 0)
HUVEC (0.9)	751	650	835	8 (100.0%)	5 (60.0%, 0, 0)
IMR90 (1.7)	2344	2137	3226	53 (71.7%)	116 (59.48%, 7.76%, 0)
K562 (1.3)	2096	1942	2972	48 (83.33%)	144 (56.25%, 6.25%, 3.33E-16)
KBM7 (1.2)	6278	5970	7862	78 (53.85%)	264 (42.8%, 8.33%, 1.25E-14)
NHEK (1.3)	1160	1018	1313	18 (88.89%)	28 (89.29%, 7.14%, 4.44E-16)

The sequencing depth is under each cell line name in the first column, in the unit of billion. The percentage in the second last column is the percent of motifs in a cell line identified in other cell lines. The four numbers in the last column are the number of the predicted motif pairs, the percentage of the predicted motif pairs in a cell line identified in other cell lines, the percentage of random motif pairs in a cell line identified in other cell lines, and the p-value of the number of the predicted motif pairs in a cell line identified in other cell lines, respectively.

The identified motif pairs were likely to be biologically meaningful because we did not discover any motif pair when we carried out the same procedure in random sequences (the first type of negative EP pairs). We generated the corresponding number of random sequences as the original input for each of the seven cell lines by randomly permuting the nucleotides in each original sequence. We could not identify any motif in any cell line by applying the same procedure to these random sequences in each cell line. We thus could not identify any motif pair, implying the biological significance of the identified motif pairs.

The predicted motifs also corroborated the biological significance of the identified motif pairs. Motif pairs were composed of pairs of motifs predicted in the corresponding cell line. We noticed that more than 80% of the predicted motifs were discovered in different cell lines on average (STAMP E-value was smaller than 1E-8 [49, 50]). The re-discovered motifs in multiple cell lines were not due to the shared EP pairs. We removed the shared EP pairs between every pair of GM12878, IMR90 and KBM7, which had the largest number of EP pairs. We could still find about 75% of the predicted motifs shared between every pair of the three cell lines. The independent discovery of the majority of motifs in other cell lines supported that these motifs were likely biological meaningful, which corroborated the function of the predicted motif pairs. Moreover, we also noticed that, on average, more than 55% of motifs in a cell line were similar to the annotated known motifs [33], further supporting the biological significance of the identified motif pairs in different cell lines.

The conservation of the identified motif pairs supported their biological significance as well. On average, more than 62% of motif pairs in one cell line were independently identified in other cell lines (Table 1). By randomly choosing the same number of motif pairs in each of the seven cell lines, we never had more than 10% random motif pairs discovered in other cell lines (p-value < 1.25E-14). After removing similar motif pairs (both pairs had STAMP E-value < 1E-08), we obtained 423 non-redundant motif pairs in seven cell lines. The conservation of the identified motif pairs suggests that these motif pairs were likely to be biologically meaningful.

### 3.2 The predicted motif pairs were enriched with motif pairs of interacting TFs

In addition to the above evidence that supported the predicted motif pairs, we noticed that the TFs binding to these motif pairs is likely to interact. We obtained the TFs that may bind to a motif by comparing the predicted motifs with known motifs. In this way, we obtained the predicted TF pairs for the corresponding predicted motif pairs. We then compared the predicted TF pairs with the known interacting TF pairs extracted from BioGRID [43] (Material and Methods). We found that the predicted motif pairs were significantly enriched with those of interacting TFs in BioGRID.

**Figure 2:**
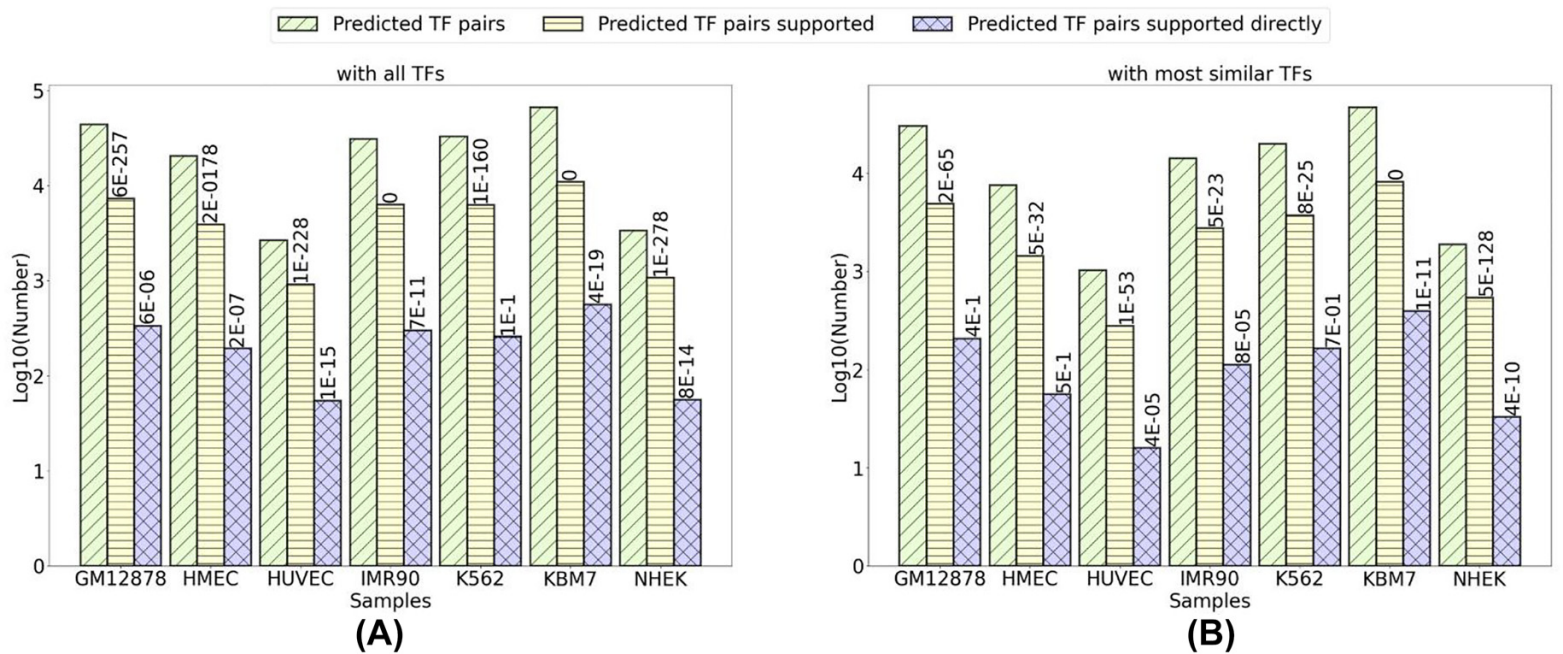
The predicted motif pairs are enriched with known interacting TF pairs.

In brief, in every cell line, we obtained TF pairs corresponding to the predicted motif pairs. Multiple TFs may bind to the same motif. We thus considered the TFs for a predicted motif in two ways: one was to include all TFs with their motifs similar to a predicted motif as the TFs of this predicted motif. The other was to consider only the TF with the most similar motif as the TF of a predicted motif (STAMP E-value < 1E-05 in both cases). In this way, we obtained two sets of TF pairs for the predicted motif pairs in every cell line (Figure 2 and Supplementary S2). We then compared each of the two sets of TF pairs with the interacting TF pairs in BioGRID. The interacting TF pairs in BioGRID interacted directly or indirectly through a third common protein (Material and Methods). We found that the predicted interacting TF pairs were significantly enriched with the known interacting TF pairs in BioGRID in almost every cell line by hypergeometric testing (Figure 2 and Supplementary S2).

Previously, Zhang et al. studied the ChIP-seq data and Hi-C data and computationally predicted the interactions of 61 TFs in GM12878 and 22 TFs in K562 [12]. We compared the predicted TF pairs in this study with theirs. There were 27 and 10 TFs in GM12878 and K562, respectively, shared by Zhang et al.’s study and this study. The two studies did not share all TFs, because certain TFs do not have sequence-specific binding motifs or a known motif. There were 55 interactions in GM12878 and four interactions in K562 involving these shared TFs identified in Zhang et al.’s study. We identified 46 of the 55 interactions in GM12878 and four of four interactions in K562 (p-value 4.0E-27 and 0, respectively).

We investigated why we did not predict the remaining nine interactions in GM12878. We found that we predict at least eight of these nine TF interactions. The motif pairs corresponding to these eight TF pairs did not satisfy the motif similarity cutoff 1E-05 when we compared the predicted motifs with the known motifs. We also examined the motif pairs that were composed of known motifs and predicted in GM12878. We could identify all of the 55 TF pairs in GM12878, including all TF pairs of the missing nine TF pairs. Moreover, we similarly compared the TF interactions predicted by Zhang et al. with the BioGRID. Zhang et al. predicted much fewer interactions, and the enrichment p-values of their predictions were much larger (Supplementary S3).

### 3.3 The predicted motif pairs were supported by EN-CODEC annotation

We compared the predicted motif pairs in GM12878 and K562 with the EN-CODEC annotation [51]. EN-CODEC did not provide motif pairs or TF pairs. Instead, it annotated TFs that bind to enhancers and promoters of individual gene based on TF-specific ChIP-seq data in GM12878 and K562. Its enhancers were defined computationally based on 10 histone markers and integrated with additional experimental evidence. The enhancers were connected to their target genes by computational methods and filtered with experimental data such as Hi-C data. Because of the computational nature of the predicted enhancers and EP pairs in EN-CODEC, together with the fact that the binding of the cofactors instead of a TF under consideration may result in the discovery of the binding of this TF instead of its cofactors in ChIP-seq, the TF-gene relation annotated in EN-CODEC may have both false positives and false negatives.

From the annotation, we defined an EN-CODEC TF pair as a pair of TFs with known motifs, in which one TF bound to enhancers and the other TF bound to promoters of the same genes for more than 30 genes. The cutoff 30 was for consistency, as each of our predicted motif pairs above occurred in at least 30 EP pairs. We considered only TFs with known motifs because we could only compare the predicted motif pairs with TF pairs of known motifs. In this way, we obtained 1379 and 4390 EN-CODEC TF pairs in GM12878 and K562, respectively, which consisted 67 TFs in GM12878 and 109 TFs in K562 (Table 2). We predicted motifs for 31 of the 67 TFs in GM12878 and 57 of the 109 TFs in K562. For motif pairs composed of these predicted motifs, more than 77% of motif pairs in GM12878 and all motif pairs in K562 were supported by the EN-CODEC TF pairs, indicating a high precision of our predicted motif pairs. On the other hand, fewer than 12% of EN-CODEC TF pairs were supported by our motif pairs.

**Table 2:** EP motif pair comparison with EN-CODEC.

Cell line	Method	% Predicted motif pairs shared with EN-CODEC	% TF pairs in EN-CODEC identified
GM12878	Based on all TFs	64/75 = 85.33%	87/1379 = 6.31%
	Based on unique TFs	51/66 = 77.27%	50/1379 = 3.63%
K562	Based on all TFs	25/25 = 100.00%	490/4390 = 11.16%
	Based on unique TFs	22/22 = 100.00%	237/4390 = 5.40%

‘Based on all TFs’ is the result based on all TFs with their motifs similar to each predicted motif (STAMP E-value < 1E-05). ‘Based on unique TF’ is the result based on the TF with its motif most similar to each predicted motif (STAMP E-value < 1E-05).

The much lower percentage of EN-CODEC TF pairs were supported, likely due to the large percentage of false positive EN-CODEC TF pairs we obtained above. Here we had only 67 TFs in GM12878 and 109 TFs in K562, while we had 1379 TF pairs in GM12878 and 4390 TF pairs in K560 (Table 2). In other words, more than 62 and 74% of all possible TF pairs regulated more than 30 genes, which was highly unlikely, indicating that the cofactors in ChIP-seq data may have biased the defined TF-gene relation in EN-CODEC. In fact, Zhang et al. integrated the same Hi-C and TF-specific ChIP-seq data in GM12878 and K562 and obtained much fewer TF pairs. Moreover, we could only identify 77 motif pairs in GM12878 and 490 motif pairs in K562 with known motifs of these TFs by the aforementioned ChIPModule analyses. The much smaller number of motif pairs identified by ChIPModule suggested that the majority of the defined EN-CODEC TF pairs did not occur in EP pairs of enough genes to be statistically significant. In other words, although we may have missed certain motif pairs that contribute to EP interactions, at least more than 77% of the predicted motif pairs were likely biologically meaningful.

### 3.4 The predicted motif pairs can help to distinguish positive EP pairs from negative ones

Since the predicted motif pairs were likely to be biologically meaningful, we tested whether they could help to distinguish positive EP pairs from negative ones (Material and Methods). We found that the predicted motif pairs separated the positive EP pairs from the first two types of negative EP pairs well and reasonably distinguished the positive EP pairs from the third type of negative EP pairs (Table 3). All had an F1 score larger than 0.66.

**Table 3:** The accuracy of motif pairs in distinguishing positive EP pairs from three types of negative EP pairs based on lasso.

Cell line	1st type	2nd type	3rd type	#Selected motif pairs	% Selected motif pairs shared
GM12878	(0.91, 0.92, 0.92)	(0.86, 0.76, 0.80)	(0.69, 0.87, 0.77)	(78, 96, 70)	43/70 = 61.43%
HMEC	(0.90, 0.90, 0.90)	(0.85, 0.72, 0.78)	(0.52, 0.99, 0.68)	(66, 58, 36)	26/36 = 72.22%
HUVEC	(0.83, 0.88, 0.85)	(0.67, 0.79, 0.70)	(0.51, 1.00, 0.67)	(5, 5, 5)	5/5 = 100.00%
IMR90	(0.91, 0.92, 0.92)	(0.91, 0.79, 0.84)	(0.50, 0.99, 0.67)	(56, 86, 43)	18/43 = 41.86%
K562	(0.91, 0.90, 0.91)	(0.87, 0.75, 0.81)	(0.50, 0.97, 0.66)	(71, 102, 53)	25/53 = 47.17%
KBM7	(0.91, 0.89, 0.9)	(0.90, 0.72, 0.80)	(0.59, 0.90, 0.71)	(107, 108, 53)	16/17 = 94.12%
NHEK	(0.89, 0.90, 0.89)	(0.65, 0.78, 0.70)	(0.51, 0.99, 0.67)	(23, 24, 17)	16/40 = 40.00%
Average	(0.89, 0.90, 0.90)	(0.82, 0.76, 0.78)	(0.55, 0.96, 0.69)	(58,68,40)	55.46%

The three numbers from the 2nd column to the 4th column are the precision, recall and F1 score. The second last column is the number of motif pairs selected by lasso in distinguishing positives from negatives for the three types of negatives in order. The last column shows the percentage of the selected motif pairs based on the third type of negatives by lasso in multiple cell lines.

We tried to determine how well the identified motif pairs could differentiate the positive EP pairs from the first two types of negative ones (Material and Methods). These two types of negative ones were “false” EP pairs. We found that the predicted motif pairs told the positive EP pairs apart from the first type of negative EP pairs with an average precision of 0.89 and a recall of 0.90 in individual cell lines in 10-fold cross-validation. Similarly, on average, the predicted motif pairs distinguished the positive EP pairs from the second type of negative EP pairs with an average precision of 0.82 and a recall of 0.76 in the 10-fold cross-validation (Table 3).

We also studied how well the predicted motif pairs separated the positive EP pairs from the third type of negative EP pairs. In the 10-fold cross-validation, the precision in all cell lines was from 0.50 to 0.69, while the recall was from 0.87 to 1 (Table 3). The much-reduced precision was likely because the number of negative EP pairs was much larger than that of positive EP pairs (Supplementary S1). We also noticed that the F1 score was decreasing from the first type of negatives to the third type of negatives, suggesting that it was more difficult to distinguish positive EP pairs from the third type of negatives than that from the first type of negatives. However, the F1 score was still above 0.66, indicating that the predicted motif pairs could distinguish the true EP interactions from the false ones. In total, lasso selected 5 to 70 motif pairs in a cell line, corresponding to 147 non-redundant motif pairs (Table 3). There were 30 motif pairs selected independently in at least two different cell lines.

We studied whether the predicted motif pairs in one cell line could distinguish the positive EP pairs from the third type of negative EP pairs in another cell line. The identified motif pairs in one cell line had similar precision and recall to distinguish the positive EP pairs from the third type of negative EP pairs in every other cell line to the predicted motif pairs from the corresponding cell line. This suggested that a large proportion of the predicted motif pairs in one cell line were likely to be conserved in another cell line. In other words, the predicted motif pairs represented conserved mechanisms across cell lines. We noticed that different cell lines shared the majority of the predicted motif pairs (Table 1) and the majority of selected motif pairs used to distinguish positive EP pairs from the third type of negative EP pairs (Table 3).

### 3.5 The selected motif pairs are likely to contribute to EP interactions

**Figure 3:**
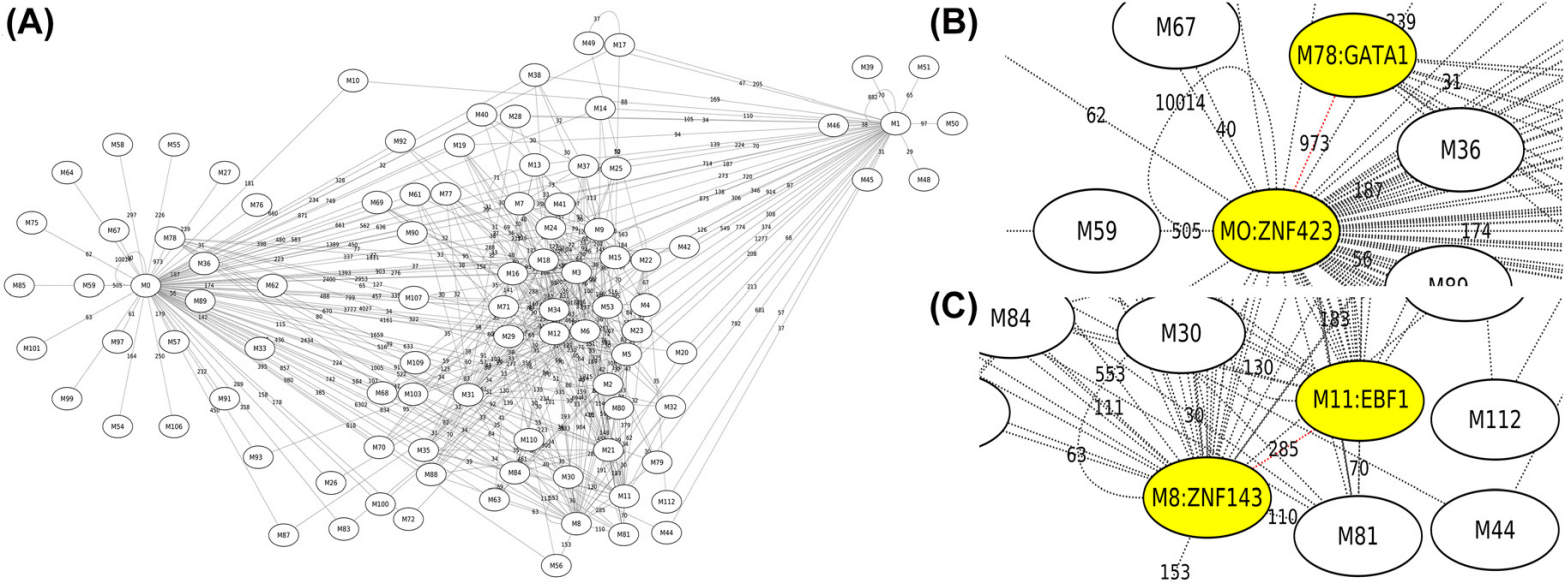
(A) The 423 motif pairs discovered in a network. (B) The TF pair GATA1-ZNF423 in the network. (C) The TF pair EBF1-ZNF143 in the network.

We studied whether the selected motif pairs from the predicted 423 motif pairs contribute to EP interactions (Figure 3A). Starting from the 147 selected motif pairs above, we identified pairs of TFs with their motifs similar to the selected motif pairs (STAMP E-value < 1E-5). We could identify TF pairs for 72 of the 147 selected motif pairs above and 19 of the 30 motif pairs selected in multiple cell lines. For 64 of the 72 selected motif pairs and 18 of the 19 selected conserved motif pairs, their corresponding pairs of TFs interacted in BioGRID. At least 45 of the 72 pairs and 14 of the 19 pairs were shown to contribute to EP interactions in literature, among which 40 of the 72 pairs and 14 of the 19 pairs are supported by BioGRID (Supplementary S4). We provided two examples of the TF pairs corresponding to these selected motif pairs in the following. The remaining motif pairs and their functional support were in Supplementary S4.

An example of a novel motif pair selected is for the TF pair GATA1-ZNF423 (Figure 3B). GATA1 is known to bind to distal regions and physically interacts with ZFPM1 in the beta-major globin promoter [52]. Like ZFPM1, ZNF521, a paralog of ZNF423 that shares 65% of homology with ZNF423, is known to have a functional NuRD sequence at the N-terminal [53, 54]. Moreover, ZNF521 modulates erythroid cell differentiation through direct binding with GATA1 [55]. It is thus evident that GATA1-ZNF423 interaction is likely to facilitate EP interaction, which may be through the GATA1 interaction with the NuRD sequence at the N-terminal of ZNF423.

Here is another novel motif pair that may facilitate EP interactions. This selected motif pair is for the TF pair EBF1-ZNF143 (Figure 3C). In vertebrates, the EBF1 is demonstrated to have the role of controlling the higher-order chromatin structure [56]. ZNF143 is known to preferentially occupy anchors of chromatin interactions connecting enhancers and promoters [57]. Moreover, EBF1, ZNF143, and RAD21 have a three-way interaction in GM12878 [56]. It is thus likely that the interaction of EBF1-ZNF143 may contribute to EP interactions [22].

## 4 Discussion

We *de novo* identified 423 motif pairs in interacting EP pairs. These motif pairs were likely to be biologically meaningful because they were statistically significant, conserved across cell lines, enriched with motif pairs of known interacting TFs, and so on. We also demonstrated that the predicted motif pairs could help to distinguish positive EP pairs from negative ones. We provided the developed EPmotifPair pipeline, the predicted motifs, motif pairs, and other related information about these motifs and motif pairs at https://doi.org/10.6084/m9.figshare.14192000.

The above analysis was based on *de novo* predicted motifs and motif pairs. We also identified 1183 motif pairs in interacting EP pairs with known motifs with the ChIPModule tool [58] (Supplementary S5). We found that most identified motif pairs based on known motifs were similar to those *de novo* predicted ones in the corresponding cell lines. For instance, in KBM7, 94% of the identified motif pairs based on known motifs were similar to the *de novo* predicted motif pairs. A small fraction of the motif pairs based on known motifs were not discovered in the *de novo* predicted motif pairs, likely due to the STAMP E-value cutoff 1E-05 used.

We noticed that more than 55% of predicted motifs were similar to known motifs in one cell line. We also observed that more than 80% of the predicted motifs in one cell line were usually identified in other cell lines. In addition, we studied whether the predicted motifs preferred to occur in enhancers and promoters (Supplementary S6). Without considering the sequence length difference between enhancers and promoters, almost all motifs preferred to occur in promoters in all cell lines. When we considered the sequence length difference, where on average the promoters were three times longer than the enhancers, there was barely any motif preferring promoters to enhancers. Therefore, the majority of motifs occurred in both enhancers and promoters, with more frequent occurrence of their binding sites in enhancers.

We also checked whether homogeneous motif pairs significantly occurred in both enhancers and promoters, such as the aforementioned CTCF-CTCF motif pair, and the YY1-YY1 motif pair (Material and Methods). If we considered the sequence lengths, 78.6–93.1% motifs could form homogenous motif pairs that significantly co-occurred in positive EP pairs, including the CTCF-CTCF motif pair in six of the seven cell lines and the YY1-YY1 motif pairs in five of the seven cell line. Even if we did not consider the sequence length, we still could identify 13, 5, and 158 motifs that could form homogeneous motif pairs in GM12878, HMEC and KBM7. In this case, CTCF was still found in HMEC. If we lower the STAMP E-value cutoff when comparing the predicted motifs with known motifs, the predicted motifs similar to CTCF and YY1 were found in GM12878 and KBM7. We provided two lists of homogeneous motif pairs based on the two different considerations at https://doi.org/10.6084/m9.figshare.14192000 for future validation studies.

Several directions may help to understand EP motif pairs better. First, although the identified motif pairs are likely to be useful in predicting EP interactions, they should be integrated with other features used previously [17, 22, 24] to fulfill their potentials. Second, a more comprehensive collection of enhancers and their condition-specific activity may improve the quality of the predicted motif pairs. The number of enhancers we used is relatively small compared with the collected enhancers in other resources [59, 60]. Third, with more annotated known motifs, it may be better to discover motif pairs directly from known motifs. We look forward to further exploring the EP motifs and their contribution to the interaction of the EP pairs.

## Supplementary Material

Supplementary Material Details
