# Supplementary material for: A systematic study of motif pairs that may facilitate enhancer–promoter interactions
Source: J Integr Bioinform. 2022 Feb 7;19(1):20210038. doi: 10.1515/jib-2021-0038 (PMC9069648; doi:10.1515/jib-2021-0038)
Supplement: Supplementary file 1 — Supplementary Material Details [file j_jib-2021-0038_suppl.docx]

**Supplementary S1 Table. Basic information about the data used in the paper.**

| **cell line** | **sequencing depth**  **(million)** | **#enhancers** | **#promoters** | **#enhancer length** | **#promoter length** | **#positive EP pairs** | **#3rd type negative EP pairs** |
| --- | --- | --- | --- | --- | --- | --- | --- |
| GM12878 | 15112.0 | 2731 | 2171 | 372 | 1100 | 3688 | 28458 |
| HMEC | 1068.0 | 1761 | 1713 | 370 | 1100 | 2157 | 10719 |
| HUVEC | 892.8 | 751 | 650 | 382 | 1100 | 835 | 4966 |
| IMR90 | 1683.1 | 2344 | 2137 | 381 | 1100 | 3226 | 8859 |
| K562 | 1366.2 | 2096 | 1942 | 367 | 1100 | 2972 | 9666 |
| KBM7 | 1247.9 | 6278 | 5970 | 320 | 1100 | 7862 | 56787 |
| NHEK | 1347.5 | 1160 | 1018 | 372 | 1100 | 1313 | 5022 |

**Supplementary S2 Table. Comparison of the predicted TF interaction with known ones in BioGRID.**

| **cell line** | **#predicted TFs supported by BioGRID** | **#predicted direct TFs pairs supported by BioGRID** | **P-value of #predicted direct TF pairs supported by BioGRID** | **#predicted TF pairs supported by BioGRID** | **P-value of # TF pairs supported by BioGRID** |
| --- | --- | --- | --- | --- | --- |
| GM12878 | 299  (265) | 335  (208) | 5.7E-06  (4.4E-01) | 7252  (4868) | 6.4E-257  (1.5E-65) |
| HMEC | 213  (139) | 194  (56) | 2.4E-07  (5.0E-01) | 3902  (1430) | 6.1E-178  (4.7E-32) |
| HUVEC | 73  (44) | 55  (16) | 1.13E-15  (4.1E-05) | 912  (277) | 8.66E-228  (1.3E-53) |
| IMR90 | 262  (205) | 297  (113) | 7.0E-11  (8.2E-01) | 6303  (2753) | 0  (5.3E-23) |
| K562 | 285  (241) | 258  (164) | 1.0E-01  (6.9E-01) | 6207  (3733) | 1.2E-160  (8.1E-25) |
| KBM7 | 359  (307) | 557  (392) | 4.4E-19  (1.19E-11) | 10876  (8179) | 0  (0) |
| NHEK | 78  (57) | 56  (33) | 7.5E-14  (4.0E-10) | 1071  (536) | 1.2E-278  (4.5E-128) |

In each entry, the information in order is the result based on all TFs for each predicted motif with STAMP cutoff 1E-05, and the result based on the most similar TF for each predicted motif with STAMP cutoff 1E-05. The p-value in 4^th^ and 6^th^ column is calculated based on hypergeometric testing. There are 1520 TFs in BioGRID, which are supported by GO. And there are 6820 TF pairs in BioGRID based on the 1520 TFs

**Supplementary S3 Table. Comparison between Zhang et al.’s study with BioGRID**

| **cell line** | **#predicted TFs supported by BioGRID** | **# predicted direct TFs pairs supported by BioGRID** | **P-value of # predicted direct TF pairs supported by BioGRID** | **#predicted TF pairs supported by BioGRID** | **P-value of # TF pairs supported by BioGRID** |
| --- | --- | --- | --- | --- | --- |
| GM12878 | 61 | 25 | 5.8E-05 | 155 | 1 |
| K562 | 22 | 4 | 1.3E-2 | 34 | 3.0E-2 |

**Supplementary S4 Table. TF pairs for 72 selected motif pairs by LASSO**

| **motif pairs** | **known TF pairs** | **supported by BioGRID** | **supported by reference** | **supported(BioGRID or literature)** |
| --- | --- | --- | --- | --- |
| M2--M36 | ZBTB7A--ZNF274,SP1--ZNF274,SP2--ZNF274,EGR1--ZNF274 | N | [[29]](https://www.ncbi.nlm.nih.gov/pmc/articles/PMC4910881/) | literature |
| M0--M1 | EGR1--FOXM1,FOXK1--KLF6,FOXM1--SP1,FOXO1--SP2,FOXK1--SP3,FOXO1--SP3,FOXK1--SP2 | Y | [[29]](https://www.ncbi.nlm.nih.gov/pmc/articles/PMC4910881/) [[30]](https://pubmed.ncbi.nlm.nih.gov/33160328/) | Both |
| M1--M2 | E2F6--MAZ,EGR1--MAZ,E2F4--ZNF263,E2F6--ZNF263,E2F4--MAZ,SP1--ZNF263,E2F1--MAZ,MAZ--SP1,MAZ--ZBTB7A,MAZ--SP2,EGR1--ZNF263,ZBTB7A--ZNF263,SP2--ZNF263 | Y | [[12]](https://www.nature.com/articles/ncomms12249) [[28]](https://pubmed.ncbi.nlm.nih.gov/26411866/) [[29]](https://www.ncbi.nlm.nih.gov/pmc/articles/PMC4910881/) | Both |
| M1--M8 | NA | N | NA | NA |
| M1--M30 | EBF1--EGR1,EBF1--SP1,EBF1--PAX5 | Y | [[12]](https://www.nature.com/articles/ncomms12249) [[29]](https://www.ncbi.nlm.nih.gov/pmc/articles/PMC4910881/) | Both |
| M0--M37 | NA | N | NA | NA |
| M0--M13 | NA | Y | NA | BioGRID |
| M1--M1 | KLF15--SP4,SP1--SP2,E2F6--SP3,E2F6--SP2,E2F1--E2F6,SP2--TAL1,EGR1--SP1,E2F4--EGR1,E2F3--SP3,E2F3--KLF6,E2F4--ZBTB7A,E2F6--ZBTB7A,SP1--TAL1,KLF4--SP1,KLF15--SP2,E2F6--EGR1,KLF15--KLF6,E2F6--KLF6,EGR1--ZBTB7A,SP2--ZBTB7A,E2F1--E2F4,E2F6--SP1,KLF15--SP3,EGR1--TAL1,SP1--ZBTB7A,EGR1--SP2,E2F4--E2F6,TAL1--ZBTB7A,E2F4--SP2,E2F4--SP1,SP1--SP3 | Y | [[27]](https://www.pnas.org/content/114/25/E4914) [[28]](https://pubmed.ncbi.nlm.nih.gov/26411866/) [[29]](https://www.ncbi.nlm.nih.gov/pmc/articles/PMC4910881/) [[30]](https://pubmed.ncbi.nlm.nih.gov/33160328/) | Both |
| M30--M7 | EBF1--MAZ | N | [[29]](https://www.ncbi.nlm.nih.gov/pmc/articles/PMC4910881/) | literature |
| M13--M7 | EGR1--MAZ,SP1--ZNF263,MAZ--SP1,MAZ--ZBTB7A,MAZ--SP2,EGR1--ZNF263,ZBTB7A--ZNF263,SP2--ZNF263 | Y | [[12]](https://www.nature.com/articles/ncomms12249) [[28]](https://pubmed.ncbi.nlm.nih.gov/26411866/) [[29]](https://www.ncbi.nlm.nih.gov/pmc/articles/PMC4910881/) | Both |
| M1--M11 | NR2C2--SP1,ETS1--SP2,ETS1--ZBTB7A,GABPA--SP2,KLF15--SP4,EHF--SP3,E2F4--ELK1,SP1--SP2,E2F6--SP3,E2F1--ELK4,EGR1--SP1,EHF--SP2,ELF2--KLF15,ELK1--SP1,GABPA--SP3,E2F3--SP3,EGR1--ELF1,E2F4--ELK4,KLF4--SP1,E2F1--GABPA,E2F6--ELK1,ELK1--SP2,E2F6--NR2C2,E2F4--ELF1,E2F4--ETS1,E2F6--ELK4,NR2C2--SP2,E2F6--GABPA,ETS1--SP1,ELF1--ZBTB7A,GABPA--ZBTB7A,E2F6--ELF1,EBF1--EGR1,EGR1--GABPA,EGR1--NR2C2,GABPA--SP1,EGR1--ELK1,ELF1--SP1,ELF1--SP2,NR2C2--ZBTB7A,EBF1--SP1,E2F6--SP1,KLF15--SP3,E2F4--NR2C2,SP1--ZBTB7A,ELK1--ZBTB7A,E2F4--GABPA,E2F6--ETS1,EGR1--ETS1,E2F1--ELK1,E2F4--SP1,SP1--SP3,E2F1--NR2C2 | Y | [[12]](https://www.nature.com/articles/ncomms12249) [[27]](https://www.pnas.org/content/114/25/E4914) [[28]](https://pubmed.ncbi.nlm.nih.gov/26411866/) [[29]](https://www.ncbi.nlm.nih.gov/pmc/articles/PMC4910881/) [[30]](https://pubmed.ncbi.nlm.nih.gov/33160328/) | Both |
| M1--M14 | MAFK--SP2,ATF1--EGR1,ATF3--E2F6,MAFK--ZBTB7A,E2F1--SMARCC1,EGR1--NFE2,E2F1--JUN,JUNB--SP1,EGR1--FOS::JUN,BACH1--ZBTB7A,FOXK1--SP2,E2F6--JUNB,EGR1--JUND,E2F1--SMARCC2,FOS::JUN--SP2,MAFF--ZBTB7A,BATF--EGR1,FOS--SP1,E2F6--FOSL2,E2F6--FOSL1,JUND--SP1,CREB1--SP2,EGR1--FOSL2,BATF--E2F4,E2F6--FOS::JUN,EGR1--MAFK,ATF1--E2F4,EGR1--JUNB,NFE2--SP1,FOS::JUN--SP1,BATF--SP1,E2F6--MAFF,E2F4--FOS,FOS--ZBTB7A,EGR1--MAFF,EGR1--JUN,EGR1--FOS,E2F6--NFE2,E2F6--JUN,E2F6--MAFK,FOSL1--SP1,MAFK--SP1,NFE2--SP2,FOSL1--SP2,JUND--ZBTB7A,E2F4--NFE2,E2F6--SMARCC1,FOXK1--SP3,EGR1--FOSL1,ATF3--SP2,E2F4--JUN,JUN--SP2,JUN--SP1,BACH1--E2F6,FOXK1--KLF6,CREB1--E2F6,E2F6--FOS,BACH1--SP1,E2F4--MAFF,CREB1--SP1,E2F6--JUND,BACH1::MAFK--SP2,E2F4--FOSL2,JUNB--SP2,CREB1--ZBTB7A,E2F4--SMARCC1,FOS--SP2,BACH1--E2F4,ATF1--ZBTB7A,E2F4--JUND,MAFF--SP2,ATF1--SP1,FOSL1--ZBTB7A,E2F4--NFE2L2,BACH1--EGR1,BACH1::MAFK--EGR1,NFE2--ZBTB7A,JUN--ZBTB7A,ATF3--E2F4,CREB1--E2F4,ATF1--E2F6,CREB1--EGR1,ATF1--SP2,EGR1--NFE2L2,ATF3--SP1,E2F4--MAFK,BACH1::MAFK--E2F6,FOSL2--SP2,BACH1::MAFK--SP1,E2F1--MAFK,E2F1--JUND,E2F1--FOS,E2F4--SMARCC2,E2F4--JUNB,BACH1--SP2,ATF3--EGR1,BACH1::MAFK--E2F4,JUNB--ZBTB7A,E2F6--NFE2L2,FOSL2--SP1,E2F6--SMARCC2,E2F4--FOS::JUN,ATF3--ZBTB7A,MAFF--SP1,E2F4--FOSL1,JUND--SP2 | Y | [[12]](https://www.nature.com/articles/ncomms12249) [[28]](https://pubmed.ncbi.nlm.nih.gov/26411866/) [[29]](https://www.ncbi.nlm.nih.gov/pmc/articles/PMC4910881/) [[30]](https://pubmed.ncbi.nlm.nih.gov/33160328/) | Both |
| M1--M38 | FOXK1--KLF6,FOXK1--SP3,FOXK1--SP2 | Y | [[30]](https://pubmed.ncbi.nlm.nih.gov/33160328/) | Both |
| M1--M35 | SP2--ZBTB7A,SP1--ZBTB7A,EGR1--ZBTB7A | Y | [[29]](https://www.ncbi.nlm.nih.gov/pmc/articles/PMC4910881/) | Both |
| M1--M31 | ELF1--SP1,GABPA--SP3,ETS1--SP2,ELF1--SP2,ETS1--SP1,ELF1--ZBTB7A,GABPA--ZBTB7A,ETS1--ZBTB7A,GABPA--SP2,ELK1--SP1,EHF--SP3,EGR1--ELF1,ELK1--ZBTB7A,ELK1--SP2,EGR1--GABPA,EGR1--ETS1,EHF--SP2,GABPA--SP1,ELF2--KLF15,EGR1--ELK1 | Y | [[12]](https://www.nature.com/articles/ncomms12249) [[27]](https://www.pnas.org/content/114/25/E4914) [[28]](https://pubmed.ncbi.nlm.nih.gov/26411866/) [[29]](https://www.ncbi.nlm.nih.gov/pmc/articles/PMC4910881/) [[30]](https://pubmed.ncbi.nlm.nih.gov/33160328/) | Both |
| M1--M13 | E2F3--SP3,E2F3--KLF6,KLF15--SP3,KLF15--SP4,SP1--ZBTB7A,EGR1--SP2,SP1--SP2,KLF15--KLF6,KLF4--SP1,EGR1--ZBTB7A,KLF15--SP2,EGR1--SP1,SP1--SP3,SP2--ZBTB7A | Y | [[27]](https://www.pnas.org/content/114/25/E4914) [[28]](https://pubmed.ncbi.nlm.nih.gov/26411866/) [[29]](https://www.ncbi.nlm.nih.gov/pmc/articles/PMC4910881/) [[30]](https://pubmed.ncbi.nlm.nih.gov/33160328/) | Both |
| M2--M35 | SP2--ZBTB7A,SP1--ZBTB7A,EGR1--ZBTB7A | Y | [[29]](https://www.ncbi.nlm.nih.gov/pmc/articles/PMC4910881/) | Both |
| M0--M3 | NA | Y | NA | BioGRID |
| M2--M5 | EGR1--MAZ,SP1--ZNF263,MAZ--SP1,MAZ--ZBTB7A,MAZ--SP2,EGR1--ZNF263,ZBTB7A--ZNF263,SP2--ZNF263 | Y | [[12]](https://www.nature.com/articles/ncomms12249) [[28]](https://pubmed.ncbi.nlm.nih.gov/26411866/) [[29]](https://www.ncbi.nlm.nih.gov/pmc/articles/PMC4910881/) | Both |
| M1--M7 | E2F6--MAZ,EGR1--MAZ,E2F4--ZNF263,E2F6--ZNF263,E2F4--MAZ,SP1--ZNF263,E2F1--MAZ,MAZ--SP1,MAZ--ZBTB7A,MAZ--SP2,EGR1--ZNF263,ZBTB7A--ZNF263,SP2--ZNF263 | Y | [[12]](https://www.nature.com/articles/ncomms12249) [[28]](https://pubmed.ncbi.nlm.nih.gov/26411866/) [[29]](https://www.ncbi.nlm.nih.gov/pmc/articles/PMC4910881/)  . | Both |
| M1--M4 | ARID3A--ZBTB7A,ARID3A--EGR1,ARID3A--SP2,ARID3A--SP1 | Y | [[29]](https://www.ncbi.nlm.nih.gov/pmc/articles/PMC4910881/) | Both |
| M1--M5 | E2F6--MAZ,EGR1--MAZ,E2F4--ZNF263,E2F6--ZNF263,E2F4--MAZ,SP1--ZNF263,E2F1--MAZ,MAZ--SP1,MAZ--ZBTB7A,MAZ--SP2,EGR1--ZNF263,ZBTB7A--ZNF263,SP2--ZNF263 | Y | [[12]](https://www.nature.com/articles/ncomms12249) [[28]](https://pubmed.ncbi.nlm.nih.gov/26411866/) [[29]](https://www.ncbi.nlm.nih.gov/pmc/articles/PMC4910881/) | Both |
| M1--M3 | KLF4--SP1,E2F6--KLF6,E2F3--KLF6,KLF15--KLF6 | Y | [[27]](https://www.pnas.org/content/114/25/E4914) [[30]](https://pubmed.ncbi.nlm.nih.gov/33160328/) | Both |
| M4--M7 | ARID3A--MAZ,ARID3A--ZNF263 | N | [[29]](https://www.ncbi.nlm.nih.gov/pmc/articles/PMC4910881/) | literature |
| M5--M7 | MAZ--ZNF263 | N | [[29]](https://www.ncbi.nlm.nih.gov/pmc/articles/PMC4910881/) | literature |
| M1--M18 | FOXO1--TFAP2B | Y | [[30]](https://pubmed.ncbi.nlm.nih.gov/33160328/) | Both |
| M0--M40 | BCL11A--FOXM1,FOXM1--IRF4,FOXM1--SPI1 | Y | [[12]](https://www.nature.com/articles/ncomms12249) [[29]](https://www.ncbi.nlm.nih.gov/pmc/articles/PMC4910881/) | Both |
| M1--M41 | MZF1--SP3,FOXO1--SP2,FOXO1--SP3,MZF1--SP2 | Y | [[30]](https://pubmed.ncbi.nlm.nih.gov/33160328/) | Both |
| M19--M2 | IRF4--SP3,BCL11A--SP1,MAZ--SP1,MAZ--ZBTB7A,BCL11A--EGR1,IRF1--ZBTB7A,SP2--ZNF263,IRF1--SP1,EGR1--SPI1,IRF1--KLF15,MAZ--SP2,SPI1--ZBTB7A,IRF4--SP4,IRF4--SP1,IRF4--SP2,EGR1--IRF4,SP2--SPI1,EGR1--ZNF263,ZBTB7A--ZNF263,EGR1--MAZ,SP1--SPI1,SP1--ZNF263,EGR1--IRF1,IRF1--SP2 | Y | [[12]](https://www.nature.com/articles/ncomms12249) [[28]](https://pubmed.ncbi.nlm.nih.gov/26411866/) [[29]](https://www.ncbi.nlm.nih.gov/pmc/articles/PMC4910881/) [[30]](https://pubmed.ncbi.nlm.nih.gov/33160328/) | Both |
| M1--M19 | E2F4--IRF1,IRF4--SP3,BCL11A--SP1,MAZ--SP1,MAZ--ZBTB7A,E2F4--SPI1,IRF1--ZBTB7A,BCL11A--EGR1,E2F6--SPI1,SP2--ZNF263,IRF1--SP1,E2F4--IRF4,EGR1--SPI1,IRF1--KLF15,E2F6--ZNF263,E2F4--MAZ,MAZ--SP2,E2F6--MAZ,SPI1--ZBTB7A,E2F4--ZNF263,IRF4--SP4,IRF4--SP1,IRF4--SP2,EGR1--IRF4,SP2--SPI1,EGR1--ZNF263,ZBTB7A--ZNF263,EGR1--MAZ,SP1--SPI1,SP1--ZNF263,E2F6--IRF1,E2F1--MAZ,EGR1--IRF1,BCL11A--E2F4,IRF1--SP2 | Y | [[12]](https://www.nature.com/articles/ncomms12249) [[28]](https://pubmed.ncbi.nlm.nih.gov/26411866/) [[29]](https://www.ncbi.nlm.nih.gov/pmc/articles/PMC4910881/) [[30]](https://pubmed.ncbi.nlm.nih.gov/33160328/) | Both |
| M0--M21 | NA | Y | NA | BioGRID |
| M0--M22 | NA | Y | NA | BioGRID |
| M0--M42 | NA | Y | NA | BioGRID |
| M0--M50 | NA | Y | NA | BioGRID |
| M0--M10 | NA | Y | NA | BioGRID |
| M10--M23 | NA | Y | NA | BioGRID |
| M1--M10 | NA | N | NA | NA |
| M22--M47 | GATA2--ZNF274,GATA1--ZNF274 | N | [[29]](https://www.ncbi.nlm.nih.gov/pmc/articles/PMC4910881/) | literature |
| M10--M50 | SP1--ZNF263,MAZ--SP1,MAZ--SP2,EGR1--ZNF263,EGR1--MAZ,SP2--ZNF263 | Y | [[12]](https://www.nature.com/articles/ncomms12249) [[28]](https://pubmed.ncbi.nlm.nih.gov/26411866/) [[29]](https://www.ncbi.nlm.nih.gov/pmc/articles/PMC4910881/) | Both |
| M1--M50 | SP1--TAL1,EGR1--TAL1,SP1--TCF3,SP1--TCF12,EGR1--TCF12,SP2--TAL1,EGR1--TCF3 | Y | [[12]](https://www.nature.com/articles/ncomms12249) [[29]](https://www.ncbi.nlm.nih.gov/pmc/articles/PMC4910881/) | Both |
| M3--M50 | EGR1--FOXM1,FOXM1--SP1,FOXO1--SP2,KLF4--SP1,FOXK1--SP3,FOXO1--SP3,FOXK1--SP2 | Y | [[27]](https://www.pnas.org/content/114/25/E4914) [[29]](https://www.ncbi.nlm.nih.gov/pmc/articles/PMC4910881/) [[30]](https://pubmed.ncbi.nlm.nih.gov/33160328/) | Both |
| M0--M18 | NA | Y | NA | BioGRID |
| M25--M26 | TFAP2A--ZNF143,SMARCC2--TFAP2A,ELK1--TFAP2C,SMARCC2--TFAP2C,ELK4--TFAP2C,GABPA--TFAP2C,TFAP2C--ZNF143,GABPA--TFAP2A,ELK1--TFAP2A,EBF1--GABPA,EBF1--ETS1,SMARCC1--TFAP2A,EBF1--SIX5,SMARCC1--TFAP2C,EBF1--IKZF1,EBF1--ZNF143,ELK4--TFAP2A,EBF1--ELK1 | Y | [[12]](https://www.nature.com/articles/ncomms12249) [[29]](https://www.ncbi.nlm.nih.gov/pmc/articles/PMC4910881/) | Both |
| M1--M25 | ETS1--SP2,ETS1--ZBTB7A,GABPA--SP2,EGR1--SIX5,ELK1--SP1,EGR1--ZNF143,GABPA--SP3,SIX5--SP1,SIX5--ZBTB7A,ELK1--SP2,SP1--ZNF143,SP2--ZNF143,ETS1--SP1,GABPA--ZBTB7A,IKZF1--SP1,EGR1--GABPA,ZBTB7A--ZNF143,GABPA--SP1,EGR1--ELK1,SIX5--SP2,EGR1--IKZF1,ELK1--ZBTB7A,EGR1--ETS1 | Y | [[12]](https://www.nature.com/articles/ncomms12249) [[27]](https://www.pnas.org/content/114/25/E4914) [[28]](https://pubmed.ncbi.nlm.nih.gov/26411866/) [[29]](https://www.ncbi.nlm.nih.gov/pmc/articles/PMC4910881/) | Both |
| M1--M54 | SP1--TAL1,EGR1--TAL1,CTCF--TAL1,CTCFL--TAL1,TAL1--ZBTB7A,SP2--TAL1 | Y | [[29]](https://www.ncbi.nlm.nih.gov/pmc/articles/PMC4910881/) | Both |
| M0--M53 | NA | Y | NA | BioGRID |
| M18--M53 | NA | Y | NA | BioGRID |
| M15--M18 | NA | Y | NA | BioGRID |
| M1--M26 | NA | Y | NA | BioGRID |
| M1--M15 | E2F3--SP3,KLF15--SP3,KLF15--SP4,SP1--ZBTB7A,EGR1--SP2,SP1--SP2,KLF4--SP1,KLF15--SP2,EGR1--SP1,SP1--SP3,SP2--ZBTB7A | Y | [[12]](https://www.nature.com/articles/ncomms12249) [[27]](https://www.pnas.org/content/114/25/E4914) [[28]](https://pubmed.ncbi.nlm.nih.gov/26411866/) [[29]](https://www.ncbi.nlm.nih.gov/pmc/articles/PMC4910881/) | Both |
| M19--M26 | EBF1--RELA | Y | [[12]](https://www.nature.com/articles/ncomms12249) | Both |
| M1--M22 | MZF1--SP3,FOXO1--SP2,FOXO1--SP3,MZF1--SP2 | Y | [[30]](https://pubmed.ncbi.nlm.nih.gov/33160328/) | Both |
| M19--M54 | CTCF--RELA | Y | [[29]](https://www.ncbi.nlm.nih.gov/pmc/articles/PMC4910881/) | Both |
| M1--M21 | EGR1--TCF12,SP1--TCF3,SP1--TCF12,MAZ--TCF3,MAZ--TCF12,EGR1--TCF3 | Y | [[12]](https://www.nature.com/articles/ncomms12249) [[29]](https://www.ncbi.nlm.nih.gov/pmc/articles/PMC4910881/) | Both |
| M18--M25 | TFAP2A--ZNF143,ELK1--TFAP2C,SMARCC2--TFAP2C,ELK4--TFAP2C,GABPA--TFAP2C,TFAP2C--ZNF143,GABPA--TFAP2A,ELK1--TFAP2A,SMARCC1--TFAP2A,SMARCC2--TFAP2A,SMARCC1--TFAP2C,ELK4--TFAP2A | Y | [[29]](https://www.ncbi.nlm.nih.gov/pmc/articles/PMC4910881/) | Both |
| M0--M14 | NA | Y | NA | BioGRID |
| M13--M58 | MZF1--SP3,FOXO1--SP2,FOXO1--SP3,MZF1--SP2 | Y | [[30]](https://pubmed.ncbi.nlm.nih.gov/33160328/) | Both |
| M0--M27 | NA | Y | NA | BioGRID |
| M0--M8 | NA | Y | NA | BioGRID |
| M0--M62 | NA | Y | NA | BioGRID |
| M0--M64 | NA | Y | NA | BioGRID |
| M0--M61 | NA | Y | NA | BioGRID |
| M0--M59 | NA | Y | NA | BioGRID |
| M0--M11 | NA | Y | NA | BioGRID |
| M3--M8 | NA | Y | NA | BioGRID |
| M26--M8 | EBF1--PAX5,IKZF1--PAX5 | Y | [[12]](https://www.nature.com/articles/ncomms12249) [[29]](https://www.ncbi.nlm.nih.gov/pmc/articles/PMC4910881/) | Both |
| M61--M8 | NA | Y | NA | BioGRID |
| M59--M61 | GATA1--ZBTB7A,GATA2--ZBTB7A | Y | [[29]](https://www.ncbi.nlm.nih.gov/pmc/articles/PMC4910881/) | Both |
| M13--M59 | GATA1--SP1,GATA2--SP1,EGR1--GATA2,GATA2--ZBTB7A,GATA1--ZBTB7A,GATA2--SP2,GATA1--SP2,EGR1--GATA1 | Y | [[29]](https://www.ncbi.nlm.nih.gov/pmc/articles/PMC4910881/) | Both |
| M21--M8 | EGR1--PAX5,MAZ--PAX5,PAX5--SP1 | Y | [[12]](https://www.nature.com/articles/ncomms12249) [[29]](https://www.ncbi.nlm.nih.gov/pmc/articles/PMC4910881/) | Both |
| M1--M69 | EGR1--FOXM1,FOXK1--KLF6,EGR1--POU2F2,POU2F2--SP1,FOXM1--SP1,FOXO1--SP2,FOXK1--SP3,FOXO1--SP3,FOXK1--SP2 | Y | [[12]](https://www.nature.com/articles/ncomms12249) [[29]](https://www.ncbi.nlm.nih.gov/pmc/articles/PMC4910881/) [[30]](https://pubmed.ncbi.nlm.nih.gov/33160328/) | Both |
| M1--M55 | NA | Y | NA | BioGRID |

**Supplementary S5 Table. Predicted motif pairs from known motifs.**

| **cell line** | **#enhancers** | **#promoters** | **# EP pairs** | **#predicted motifs (%shared)** | **#predicted motif modules** | **#predicted motif pairs** |
| --- | --- | --- | --- | --- | --- | --- |
| GM12878 | 2371 | 2171 | 3688 | 50  (96.0%) | 10425 | 444  (95.3%, 12.2%, 2.1E-14, 89.4%) |
| HMEC | 1761 | 1713 | 2157 | 31  (100.0%) | 736 | 98  (100.0%, 14.3%, 3.6E-15, 86.7%) |
| HUVEC | 751 | 650 | 835 | 10  (100.0%) | 41 | 13  (100.0%, 30.8%, 2.2E-07, 46.2%) |
| IMR90 | 2344 | 2137 | 3226 | 48  (97.9%) | 9770 | 477  (86.6%, 10.5%, 3.2E-15, 89.5%) |
| K562 | 2096 | 1942 | 2972 | 48  (97.9%) | 7480 | 376  (91.8%, 10.9%, 0, 88.0%) |
| KBM7 | 6278 | 5970 | 7862 | 74  (76.6%) | 28701 | 989  (48.6%, 13.7%, 4.8E-14, 94.0%) |
| NHEK | 1160 | 1018 | 1313 | 12  (100%) | 118 | 24  (100.0%, 16.7%, 1.4E-15, 79.2%) |

The percentage in the “#predicted motifs” column is the percent of motifs in a cell line identified in other cell lines. The four numbers in the last column are the number of the predicted motif pairs, the percent of the predicted motif pairs in a cell line identified in other cell lines, the percentage of random motif pairs in a cell line identified in other cell lines, and the p-value of the number of the predicted motif pairs in a cell line identified in other cell lines, and the percentage of motif pairs found in the de novo predicted motif pairs independent of known motifs in the paper, respectively.

**Supplementary S6 Table.** **Almost all motifs (SIOMICS) are likely to occur in both enhancers and promoters.**

| **cell line** | **# predicted motifs** | **with length** | | | **without length** | | |
| --- | --- | --- | --- | --- | --- | --- | --- |
|  |  | **%Enhancer motif** | **%Promoter motif** | **%Enhancer and Promoter motif** | **%Enhancer motif** | **%Promoter motif** | **%Enhancer and Promoter motif** |
| GM12878 | 241 | 51.5% | 2.1% | 46.4% | 0.4% | 99.2% | 0.4% |
| HMEC | 83 | 45.8% | 0% | 54.2% | 2.4% | 96.4% | 1.2% |
| HUVEC | 14 | 14.3% | 7.1% | 78.6% | 0.0% | 100.0% | 0.0% |
| IMR90 | 190 | 61.1% | 1.5% | 37.4% | 0.0% | 99.5% | 0.5% |
| K562 | 180 | 49.4% | 2.2% | 48.3% | 0.5% | 98.9% | 0.6% |
| KBM7 | 428 | 57.0% | 1.2% | 41.82% | 0.7% | 98.8% | 0.5% |
| NHEK | 29 | 31.0% | 13.8% | 55.2% | 0.0% | 100.0% | 0.0% |
